# Determining the optimal antioxidant strategy: a comparative analysis of extraction methods in *Cistus villosus* L. extracts revealed by LC-HRMS and computational modeling

**DOI:** 10.55730/1300-0152.2720

**Published:** 2024-11-12

**Authors:** Hicham BOUAKKAZ, Ebru EROL, Khedidja BENAROUS, Gülaçtı TOPÇU, Reguia MAHFOUDI, Amar DJERIDANE, Mohamed YOUSFI

**Affiliations:** 1 Fundamental Sciences Laboratory, Amar Telidji University, Laghouat, Algeria; 2 Department of Analytical Chemistry, Faculty of Pharmacy, Bezmialem Vakıf University, İstanbul, Turkiye; 3 Department of Pharmacognosy, Faculty of Pharmacy, Bezmialem Vakıf University, İstanbul, Turkiye; 4 Drug Application and Research Center (DARC), Bezmialem Vakıf University, İstanbul, Turkiye; 5 Laboratory of Applied Sciences and Didactic, Higher Normal School of Laghouat, Algeria

**Keywords:** *Cistus villous* L., antioxidants, maceration, LC-HRMS, natural health, docking

## Abstract

**Background/aim:**

*Cistus villosus* L., a beloved tea herb, contains a treasure trove of potent antioxidants. Our research unveils this ancient ally’s potential to shield against oxidative stress, a modern health threat.

**Materials and methods:**

Antioxidant assays were conducted to evaluate the potency of ethyl acetate extract. IC_50_ values were determined for the extract in various antioxidant assays. Furthermore, molecular docking was employed for the examination of the 22 compounds analyzed through LC-HRMS. Additionally, ADMET and PASS investigations were conducted to evaluate their capability as antioxidants and inhibitors of oxidant formation.

**Results:**

Each identified compound boasts a history of antioxidant prowess, building the natural defense arsenal of *C. villous*. Antioxidant assays showed the potency of the ethyl acetate fraction with an IC_50_ value of 5.30 mg/L in the DPPH• test, which is better than that of the studied standard, α-tocopherol (36.35 mg/L). The pharmacokinetic profiles demonstrated good properties for the identified compounds. The PASS study unveiled their potential as antioxidants, and molecular docking showed their effectiveness as inhibitors of oxidant formation in the human body by blocking the studied targets.

**Conclusion:**

This herb, a timeless testament to nature’s bounty, stands poised to become a powerful weapon in our fight against oxidative stress, promising a future of natural well-being.

## 1. Introduction

*Cistus villous* L. var*. undulates*, also known as *Cistus creticus* subsp. *creticus*, a member of the family Cistaceae, thrives in the nutrient-poor soils of the Mediterranean region, showing a remarkable ability to adapt. This small, dimorphic woody shrub is found seasonally along the coasts of the central-eastern Mediterranean, northern Africa, and western Asia, except for France and the Iberian Peninsula. Widely known for its therapeutic properties, *C. creticus* is commonly used as a medicinal plant and it is often consumed in tea form; it is known for its DNA-reactive capabilities (Davis 1965; Kilic et al. 2019). Traditional medicine has exploited the healing potential of plants such as *C. creticus* for centuries, using them to treat various ailments, including diarrhea, peptic ulcers, skin rashes, and urinary tract infections (Küpeli and Yesilada 2007).

Studies on *Cistus creticus* subsp. *creticus* have shed light on the physiology of its leaves and its traditional applications in folk medicine. In addition, research has confirmed its various beneficial properties, including antimicrobial, antioxidant, antitumor, nociceptive, and analgesic effects attributed to its leaf extracts (Stefi et al., 2022). Notably, these extracts show an antiproliferative effect on human prostate cells. This herb also showed selective activities against the influenza virus, due to its rich composition of bioactive secondary metabolites, including flavonoids, aromatic compounds, phenols, and tannins (Chon, 2012). Among these compounds, phenolic compounds are particularly abundant in its leaves, which confirms its distinction as a plant containing a large accumulation of phenolic compounds (Küpeli and Yesilada, 2007).

The study conducted on *C. creticus* focuses on two subspecies, *C. creticus* subsp. *enicocephalids* and *C. creticus* subsp. *cornices*, and involves investigations into their morphology, chemical composition, and genetic data. Variations were observed between the subspecies in terms of their morphology, essential oil production, volatile fraction composition, and genetic information. The objective of the study was to contribute to a more comprehensive understanding of *C. creticus* and its economic importance (Paolini et al., 2009). The investigation on *C. creticus* subsp*. creticus* aimed to establish an in vitro culture for the production of labdanum-type diterpenes with antibacterial and cytotoxic properties. Characterization of ethanol extracts from shoots and roots showed that labdanum diterpenes were predominantly present in shoot extracts. The extracts exhibited cytotoxic effects on cervix, breast, and melanoma cancer cells. A previous study was the first to report the cytotoxic effect of labdanum diterpenes from *Cistus* on cervix cells (Skorić et al., 2012). Histochemical staining revealed that trachoma’s of *C. creticus* subsp. *creticus* could produce bioactive terpenoids and phenylpropanoids. The main antibacterial compound in *C. creticus* resin was identified as ent-3*β*-acetoxy-13-epi-manoyl oxide, which showed inhibition against both gram-negative and gram-positive bacteria. This finding suggests the potential for large-scale production protocols (Skorić et al., 2022). The study documents the antibacterial activity of seven labdanum-type diterpenes, including a newly discovered natural product. Five diterpenes were isolated from the *C. creticus* resin, two of which were hemi synthetic derivatives (Kalpoutzakis et al., 1998). The investigation focuses on *Cistus creticus* subsp. *creticus*, also known as rockrose, which has a long history of traditional medicinal use and is widely distributed in Greece and the Mediterranean region. Recent interest has centered around the antiviral properties of compounds derived from its flavonoid fraction against influenza virus and HIV (Aničić et al., 2021). Despite the chemical characterization of bioactive metabolites in the leaves, the genetic basis of flavonoid biosynthesis in *Cistus* remains largely unexplored. Researchers examined flavonoid metabolism during fruit development using comparative metabolomics and transcriptomic approaches (Aničić et al., 2021).

The results of previous studies highlight the importance of *Cistus* fruit as a significant source of flavones, flavan-3-ols, and proanthocyanins, with levels decreasing as the fruit matures. The main identified proanthocyanins are B-type procyanidins and PR delphinidins, with Gallo catechol and catechin as the dominant flavan-3-ols. The expression patterns of biosynthetic genes and transcription factors throughout flower and fruit development were analyzed. The results revealed that flavonoid-biosynthetic genes are regulated during development, with transcript levels decreasing as the fruit matures. Positive correlations between targeted metabolite content and gene expression suggest that transcriptional regulation plays a role in flavonoid biosynthesis (Pateraki and Kanellis, 2010). The research suggests that leucoanthocyanidin reductase is a key enzyme in controlling flavan-3-ol formation, leading to the production of catechin and Gallo catechol as major building blocks for *Cistus* proanthocyanins. Additionally, a decrease in ethylene production during no climacteric fruit maturation is associated with the down regulation of flavonoid- and ethylene-related biosynthetic genes and transcription factors, as well as a decrease in flavonoid content (Falara et al., 2008).

A stress-induced elevation in cortisol levels triggers an acceleration in glucose metabolism, thereby leading to an increase in the production of reactive oxygen species (ROS). Consequently, this surge in ROS production can result in oxidative stress within organisms due to the incapability of effectively detoxifying the ROS or repairing the ensuing damage (Halliwell and Gutteridge, 2015). Numerous studies have presented evidence highlighting the detrimental effects of oxidation on various biomolecules including proteins, lipids, and DNA, which in turn can contribute to the development of several diseases such as diabetes, Alzheimer’s, cancer, and atherosclerosis. This phenomenon has been extensively documented in the scientific literature by researchers (Choi et al., 2019). Thus, it is widely acknowledged that antioxidants play a crucial role in mitigating the risk of a diverse range of diseases. Recent scientific investigations have yielded noteworthy findings, demonstrating the robust antioxidant activity exhibited by compounds such as flavonoids, phenolic, and terrenes that have been extracted from natural sources (Shah et al., 2014). This has been corroborated by studies (Haile, 2015; Ismail and Mauer, 2020), as well as predictions (Shabbir et al., 2023).

The present study delves into the antioxidant potential of extracts obtained via two distinct methods. Employing a battery of antioxidant assays and detailed chemical profiling to dissect and contrast their potency, we will compare their efficacy and elucidate their interactions with key human antioxidant targets. This comparative analysis will shed light on the most effective extraction method and uncover potential mechanisms of action through interaction studies with crucial human antioxidant targets.

## 2. Materials and methods

### 2.1. Materials

The plant material was collected in November 2022 from Dar, which is located 134.5 km from Laghouat city center (34°54′51.8″N 3°20′03.2″E). This plant was identified at the Fundamental Sciences Laboratory, Amar Telidji University, Laghouat, Algeria, and voucher specimen were deposited with code LSF-10-22 at our research laboratory (Bouakkaz et al., 2024a). To preserve their bioactive compounds, the collected plant specimens were subjected to a natural drying process. Air circulation was ensured in a well-ventilated space maintained at ambient temperature. Following the drying period, the plant material was meticulously ground using a pestle and mortar to obtain a fine powder for further analysis.

### 2.2. Preparation of plant extracts

**Figure 1:**
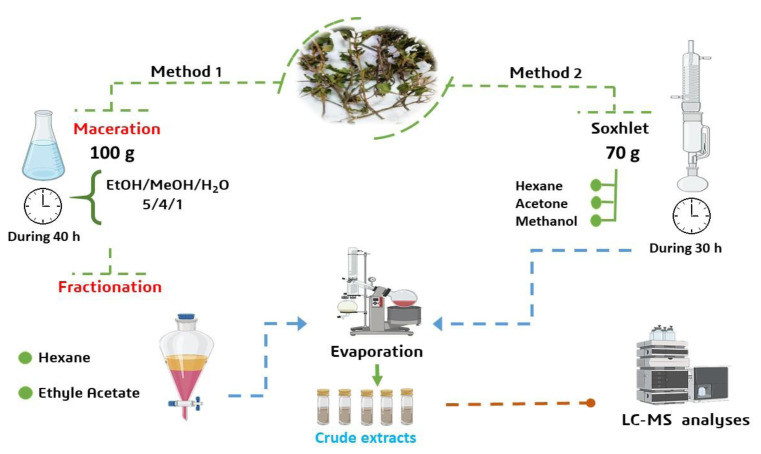
Work flow of secondary metabolites’ extraction.

In pursuit of the most effective extraction strategy for secondary metabolites, we evaluated two distinct protocols on the aerial parts of *C. villous*. Figure 1 details the meticulous procedures employed, where the total volumes of solvents used in the extraction are 250 mL for the first method for all solvents and 50 mL, 40 mL, and 10 mL for ethanol, methanol, and water, respectively, for the second method (Bouakkaz et al., 2024a and 2024b), ultimately leading to ethyl acetate (EtOAc) fraction and methanol extract (MeOH).

To standardize the names of the plant extracts prepared, we proposed the following codes:

- ECECc: Ethyl acetate fraction of *C. villous* L. var. *undulates*, obtained by maceration.
- MCECc: Methanol crude extract of *C. villous* L. var. *undulates*, obtained using a Soxhlet apparatus.

### 2.3. Profiling of secondary metabolites

Quantitative analysis of phenolic was performed utilizing a Thermo Orbit rap Q-Enactive liquid chromatography high-resolution mass spectrometer (LC-HRMS, Thermo Fisher Scientific Inc., Waltham, MA, USA) coupled with a reverse-phase C18 column (150 × 3 mm × 5 μm particle size, Trysail). A certain amount of the MeOH extract and EtOAc fraction was dissolved in 2 mL of LC-grade methanol; then samples were filtered using a syringe filter with a 0.02-μm pore size. Analysis was performed using the chromatography method described in a previous study (Erol, 2024).

In the high-resolution mode of MS analysis (HRMS), the mass spectrometer scanned ions across the m/z range from 100 to 900, generating both negative and positive ions with electrospray ionization (ESI) as the ionizing agent. The mass spectrometric analysis was configured with the following parameters: sheath gas flow rate, 45 psi; auxiliary gas flow rate, 10 psi; spray voltage, 3.80 kV; capillary temperature, 320 °C; S-lens RF level, 50. For the quantification of phenolic compounds, comparisons were made between the retention times and HRMS data of sample compounds and established reference compounds. Dihydrocapsaicin served as an internal standard for LC-HRMS measurements, mitigating repeatability issues arising from external variables, such as ionization repeatability. The quantitative analysis involved determining the phenolic content in the extracts (expressed as μg/g extracts) using a calibration curve. All the phenolic compounds used in the study were purchased and utilized as standard (Erol et al., 2024).

### 2.4. Antioxidant assays

#### 2.4.1. Free-radical scavenging activity (DPPH assay)

To assess the ability of the extracts to scavenge free radicals, the DPPH assay, as described by Blois in 1958 was employed. ECECc and MCECc were dissolved in methanol to create solutions with concentrations ranging from 6.25 to 800 μg/mL. After the addition of the DPPH solution, absorbance was measured at 517 nm following 30-min incubation at room temperature in the dark. Standard chemicals mutilated hydroxyanisole (BHA) and *α*-tocopherol (*α*-Toc) were used, with methanol serving as the control solvent. The results are expressed as the 50% inhibition concentration in μg/mL (IC_50_) based on the methodology outlined by (Yaylı et al., 2021).

The percentage of enzyme inhibition was calculated using the following formula:


$$\text{Inhibition }(\%)=\frac{\left({\text{A}}_{control}-{\text{A}}_{samples}\right)}{{\text{A}}_{control}}\times 100,$$

where

A_control_ is the absorbance of the control (without extract).
A_sample_ is the absorbance of the sample (with extract).

#### 2.4.2. ABTS cation radical scavenging activity (ABTS•+ assay)

Following the methodology outlined by Re et al. (1999) with slight modifications, the ABTS cation radical scavenging activity of the extracts was examined. ABTS^•+^ radical solution was prepared using 2,2-azino-bis-(3-ethylbenzothiazoline-6-sulfonic acid) and K_2_S_2_O_3_ to obtain 7 mm Subsequently, the extracts were evaluated for their impact on ABTS cation radical scavenging at 734 nm after 10-min incubation in the dark (Erol, 2023; Erol, 2024).

#### 2.4.3. *β-*Carotene/linoleic acid assay

The antioxidant activity of the obtained extracts was assessed using a slightly modified version of the *β*-carotene/linoleic acid assay, originally described by (Ebru, 2023), with slight modifications. In this procedure, 25 μL of linoleic acid and 200 μL of a Tween-40 emulsifier combination were added to 0.5 mg of *β*-carotene dissolved in 1 mL of chloroform. After vacuum-assisted chloroform evaporation, 100 mL of distilled water saturated with oxygen was introduced. The zero-time absorbance at 470 nm was promptly determined using a 96-well plate reader upon application of the emulsion to each tube. The emulsion system was then incubated at 50 °C for 2 h. BHA and *α*-Toc were utilized as standard antioxidants for comparative purposes.

#### 2.4.4. Cupric reducing antioxidant capacity (CUPRAC) assay

The antioxidant activity of the extracts in reducing cupric ions was evaluated with a modified procedure based on Apak et al.’s method (2007). In each well of a 96-well plate, 50 μL of 10 mm Cu (II), 50 μL of 7.5 mm neocuproine, and 60 μL of NH_4_Ac buffer (1 M, pH 7.0) solutions were added. Subsequently, the extracts, spanning the same concentration range, were introduced. The absorbance at 450 nm was measured after 1-h incubation at room temperature. To facilitate comparison, standard antioxidants BHA and *α*-Toc were utilized in the study (Karan et al., 2021).

#### 2.4.5. Ferrous ion chelating activity

The chelating activity of the extracts on Fe^2+^ was assessed by employing ferine as described by Decker and Welch in 1990, with slight modifications. In this procedure, 40 μL of 0.20 mm FeCl_2_ was added to the extract solution, prepared in DMSO and methanol to yield solutions at eight different concentrations: 6.25, 12.5, 25, 50, 100, 200, 400, and 800 μg/mL. To initiate the reaction, 80 μL of 0.5 mm ferine was introduced. Following 10-min incubation at room temperature, the mixture was measured at 562 nm. Additionally, EDTA was used as standard compound (Cherrak et al., 2016).

### 2.5. ADMET and drug-likeness evaluation

The optimization of drug dosages heavily relies on the prediction of pharmacokinetics. However, the identification of potential drug interactions and the assessment of toxicity are crucial in order to prevent setbacks during the later stages of drug development. This entire process involves the evaluation of drug absorption, distribution, metabolism, excretion, and toxicity (ADMET) within the body (Linani et al., 2023a; Linani et al., 2024; Shi et al., 2021). To ensure that our compounds possess favorable pharmacokinetic properties, we consulted the following online servers to analyze their profiles:

▪ SwissAdme^1^
▪ ADMETLab^2^
▪ ADMETSar^3^
▪ The pre-ADMET v2.0 online server^4^

**Figure 2:**
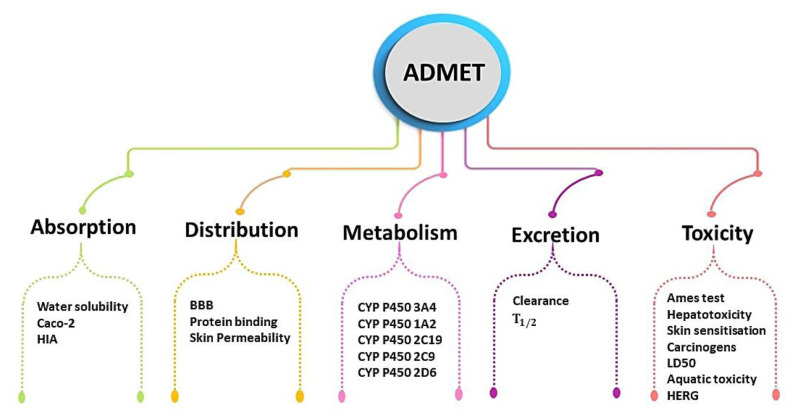
Illustration of the pharmacokinetics parameters used for ADMET analyses.

These servers enable us to predict and access data from previous in vitro and in vivo assays compiled from diverse databases. Additionally, they facilitate comprehensive access to clinical trials pertaining to small molecules and peptides along with bioassays chemical registration and analytical techniques such as nuclear magnetic resonance spectroscopy and mass spectrometry to generate predictions (Bouakkaz et al., 2024b). The employed ADMET parameters are illustrated in Figure 2.

### 2.6. Biological activity prediction (PASS)

PASS stands for the Prediction of Activity Spectra for Substances. It is a freely accessible online server utilized to forecast the biological activity of chemical compounds by analyzing their structural characteristics.^5^ We followed the same steps detailed in our previous works (Benarous et al., 2022; Linani et al., 2023a; Linani et al., 2023b; Serseg et al., 2022; Serseg et al., 2023).

### 2.7. Molecular docking study

Molecular docking is a well-developed computational technique that uses the structure of molecules to predict their interactions with each other by predicting possible binding modes and estimating binding affinity (Benarous et al., 2022). This helps in understanding and estimating molecular recognition in terms of both structure and binding energy. It is widely used in the field of drug discovery (Bellahcene et al., 2024; Linani et al., 2023a; Linani et al., 2023b). The docking is run with the software Auto Dock Vina v.1.2.0 (The Scripps Research Institute, La Jolla, CA, USA) (Serseg et al., 2023). Initially, 22 major compounds from ECECc and MCECc chemical profiles obtained from LC-HRMS results were selected as ligands. The structures of these compounds were acquired from the PubChem database^6^ and subsequently recorded in PDB format using Discovery Studio visualizer v.2016^7^ (Benarous et al., 2022; Danne et al., 2021). The 3D structure of the two targets was downloaded from the Protein Data Bank^8^ (Benarous et al., 2022), as human Keap1-Neh2 complex (PDB ID 2flu, with resolution of 1.50 Å), the BTB-Kelch substrate adaptor protein Keap1 controls steady-state levels of the bZIP transcription factor Nrf2 in response to oxidative stress. The second protein is human p22phox in combination with p47phox peptide (PDB ID 7yxw, with resolution of 2.50 Å), which is essential for NOX activation and the subsequent generation of superoxide anions and reactive oxygen species. Furthermore, a series of preparation processes were applied to each target. Firstly, the structure was modified by removing all water molecules and cocrystalized ligands through the utilization of Discovery Studio Visualizer 2016 (Kamal and Chakrabarti, 2023) The next step involves the incorporation of polar hydrogens and partial charges into the target structure using AutoDock Tools v.1.5.6. The last step consists of determining the grid box’s center and size for each protein (Table 1). The number of output conformations was set to one with 10 docking runs performed. Finally, the generated docking results were imported into Discovery Studio Visualizer 2016 (Benarous et al., 2022; Linani et al., 2023a; Linani et al., 2023b; Serseg et al., 2022; Serseg et al., 2023).

## 3. Results and discussion

### 3.1. Extraction yield

One hundred grams of plant material was extracted using the solvent mixture of ethanol, methanol, and water (5:4:1) by maceration for 35 h (method 1). In method 2, 70 g of plant material was extracted with hexane, acetone, and methanol, consecutively, using a Soxhlet apparatus for 24 h (Table 2).

### 3.2. Profiling of phenolics

In the current investigation, the ethyl acetate fraction was obtained through a meticulous liquid–liquid extraction process from the hydroalcoholic extract (EtOH: MeOH: H_2_O, 5:4:1) of the aerial part of *C. villosus* L. var*. undulatues* (method 1). In addition, we carried out the extraction of this plant material by means of the widely employed Soxhlet apparatus (method 2) with hexane, acetone, and methanol, consecutively. The evaluation and quantification of the phenolic compounds in the ethyl acetate fraction and MeOH extract, which exhibited the highest antioxidant activity, were conducted using advanced liquid chromatography coupled with high-resolution mass spectrometry (LC-HRMS).

**Figure 3:**
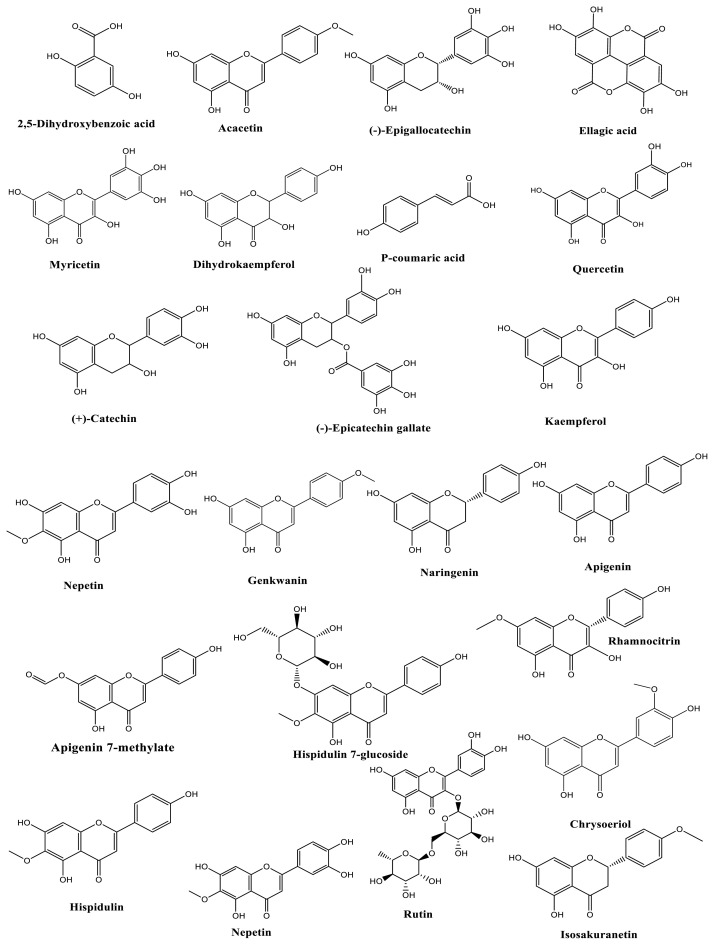
Major phenolics of *C. villosus* L. var. *undulatus*.

**Figure 4:**
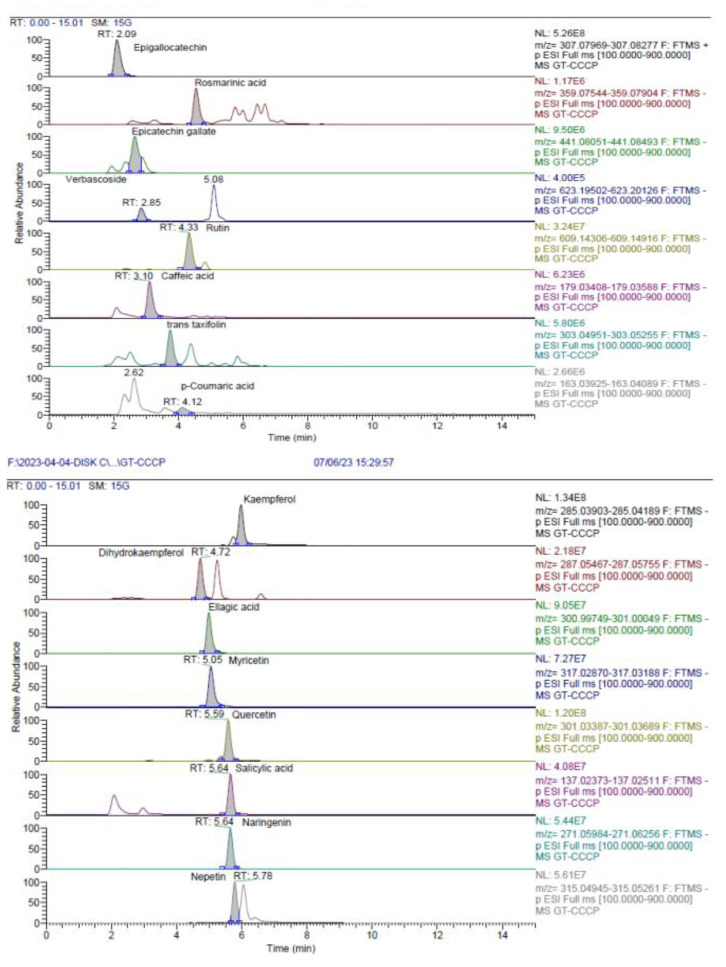
LC-HRMS chromatograms of ECECc.

**Figure 5:**
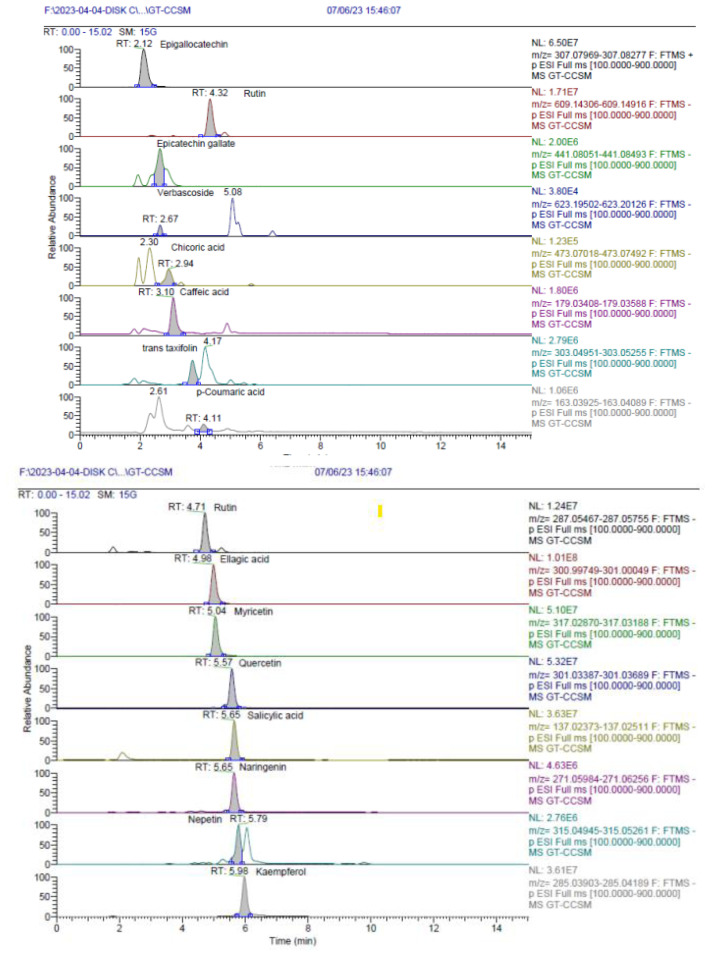
LC-HRMS chromatograms of MCECc.

A total of 32 different phenolic compounds were identified, with 28 in the MeOH extract (Table 3; Figure 3) and 29 in the EtOAc fraction (Table 4). In addition to the LC-HRMS chromatograms of both extracts (Figures 4 and 5), catechin (flavan-3-ol; (2R, 3S)-2-(3,4-dihydroxyphenyl)-3,4-dihydro-2H-chromene-3,5,7-triol) was recognized as the primary phenolic compound in the MeOH extract, while epigallocatechin was identified in the EtOAc fraction. These catechin derivatives, found abundantly in the plants belonging to the genus *Cistus*, contribute to the diverse array of bioactive compounds and phytochemicals present in these species (Maggi et al., 2016). Tea catechins have been reported to have diverse physiological functions, including activities that are antiatherogenic, antiobesity, antioxidative, and anticarcinogenic (Abd Razak et al., 2024; Farhan, 2022; Hase et al., 2001; Li et al., 2022; Sidhu et al., 2024).

In a placebo-controlled, double-blind human study, participants with mild hypercholesterolemia were given a drink containing 197 mg of heat-treated tea catechins twice a day for 12 weeks. The study revealed that the experimental group had a 5% lower serum cholesterol concentration compared to the placebo control group (Araki et al., 2005; Kawamori et al., 2003).

Moreover, kaempferol (flavonol; (3,5,7-trihydroxy-2-(4-hydroxyphenyl)-chromen-4-one)) and hispidulin (7-*O*-glucoside-(flavone; 5-hydroxy-2-(4-hydroxyphenyl)-6-methoxy-7-[(2S,4S,5S)-3,4,5-trihydroxy-6-(hydroxymethyl)oxan-2-yl]oxychromen-4-one) were identified as the secondary major compounds in the MeOH extract and EtOAc fraction, respectively (Barrajón-Catalán et al., 2011; Dou et al., 2007; Kilavik et al., 2013). Kaempferol shows potential health benefits, including cancer prevention, cardiovascular health improvement, and antiinflammatory effects, making it a promising candidate for further research and clinical exploration. Its bioavailability, antibacterial properties, and role in preventing atherosclerosis contribute to its multifaceted impact on health (Rasouli et al., 2017). In traditional and complementary medicine, it is asserted to possess properties such as antioxidant, antifungal, antiinflammatory, antimutagenic, and antineoplastic effects (Rasouli et al., 2017).

In addition, ellagic acid (polyphenol; 2,3,7,8-tetrahydroxy [1] benzopyrano[5,4,3-cde] [1] benzopyran-5,10-dione) was found in the 3rd highest amount in the MeOH extract, while rutin (flavonoid glycoside; 3′4′5′7-tetrahydroxy-3-[α-L-rhamnopyranosyl-(1→6)-β-D-glucopyranosyloxy] flavone) was identified in the EtOAc fraction.

The observed differences in compound profiles and bioactivities between the extracts can be attributed to variations in experimental parameters, such as temperature and solvent polarity. Notably, ethyl acetate fractions demonstrated superior activity and compound quality compared to methanol fractions. These results suggest that method 1 is the most effective approach for maximizing both yield and potency.

### 3.3. Antioxidant activities

**Figure 6:**
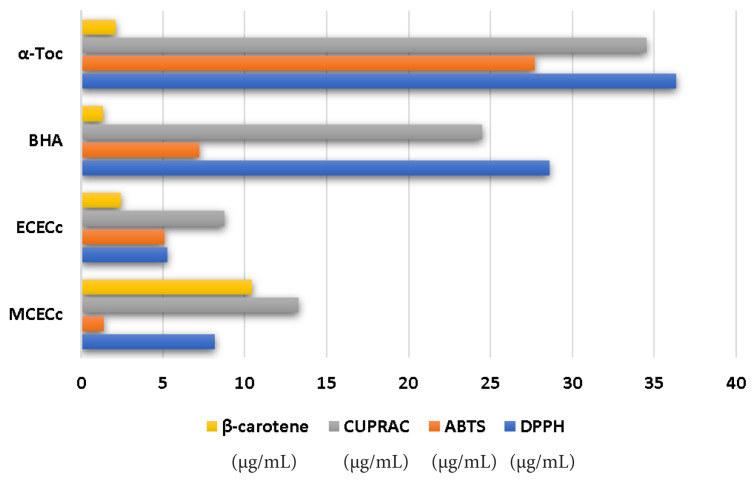
Antioxidant activity results of the MCECc and ECECc. (μg/mL)

The outcomes presented herein provide valuable insights into the antioxidative and metal chelating capabilities of different extracts derived from *C. villosus* L. var. *undulatues*. These capacities were thoroughly evaluated using a range of well-established assays, including DPPH, ABTS, CUPRAC, metal chelating, and *β*-carotene (Table 5; Figure 6). In the DPPH and ABTS assays, the MCECC and MCECc extracts exhibited significant antioxidant activities, each displaying distinct IC_50_ values. Interestingly, the ECECC extract particularly stood out in the CUPRAC test, showing an impressive IC_50_ value of 8.74 ± 0.19 mg/L, which is indicative of its ability to effectively reduce copper ions and act as a potent antioxidant. It is noteworthy that the metal chelating activity of EDTA, as evidenced by the IC_50_ value obtained from the metal chelation assay, clearly underscores its efficacy as a chelating agent. Furthermore, it is intriguing to observe that the natural extracts, namely MCECc and ECECc, demonstrate a competitive antioxidant performance when compared to synthetic antioxidants like BHT and BHA (Dinh Ngoc et al., 2021). This finding highlights the potential of these natural extracts as effective alternatives in the realm of antioxidative agents. Notably, the results obtained from the *β*-carotene test affirm the capacity of BHA and *α*-Toc to safeguard *β*-carotene from oxidation, with the MCECc also displaying a similar but distinct performance. In conclusion, the findings of the present study unequivocally establish the substantial antioxidative potential and metal-chelating capabilities possessed by the MCECc extract and ECECc fraction, thereby emphasizing the necessity of exploring natural sources in the quest for efficacious antioxidants. Additionally, it is imperative to conduct further research to elucidate the specific bioactive compounds responsible for these observed activities, as this knowledge can pave the way for potential applications in functional foods or pharmaceuticals.

The study observed the antioxidant activity of acacetin (ACA), which also presented evidence that ACA treatment can augment the oxidant/antioxidant system by increasing total antioxidant capacity (TAC) and reducing reactive oxygen species (ROS) levels (Antropova et al., 2020; Halliwell and Gutteridge, 2015). Accordingly, it can be inferred that the antioxidant activity of ACA may be attributed to its capability to eliminate free radicals and hinder lipid peroxidation. Myricetin has garnered noteworthy attention due to its potent antioxidant and free-radical scavenging characteristics, which can safeguard cells and tissues from oxidative damage (Choi et al., 2019; Laftouhi et al., 2023).

Consequently, it holds potential for various health-related applications, such as shielding skin from UV radiation and environmental stressors. (−)-Epigallocatechin gallate (EGCG) and (−)-epigallocatechin (EGC) are catechins present in green tea that are renowned for their antioxidant properties (Shah et al., 2014). Their ability to counteract free radicals and provide protection against oxidative damage has been extensively investigated, with the identification of oxidation products shedding light on the mechanisms underlying their antioxidant reactions (Haile, 2015). The role of rutin in antioxidant activity is significant as it can counteract free radicals and defend against oxidative stress (Ismail and Mauer, 2020). This is evident from its ability to inhibit lipid peroxidation and demonstrate reducing power by donating electrons to reactive free radicals (Ed-Dahmani et al., 2024; Song et al., 2021).

### 3.4. ADMET and drug-likeness evaluation

The ADMET parameters that have been anticipated have been meticulously presented within the comprehensive and informative Table 6, where a detailed analysis of these parameters has been conducted to elucidate the intriguing characteristics of the compounds and shed light on their potential challenges. In terms of absorption, all ligands exhibited a low Caco-2 cell permeability, except *p*-coumaric acid and ellagic acid, which can easily permeate these cells. Genkwanin, chrysoeriol, acacetin, rhamnocitrin, isosakuranetin, *p*-coumaric acid, hispidulin, apigenin, and naringenin achieved the optimal human intestinal absorption levels compared to the other compounds. The distribution profile shows that the blood–brain barrier penetration value of all selected compounds was low. However, they could bind to plasma proteins strongly, except rutin.

(−)-EGC, *p*-coumaric acid, and (−)-epicatechin gallate share identical metabolism profiles, which means they cannot be substrates or inhibitors for cytochrome P4503A4, 1A2, 2C19, 2C9, or 2D6. In terms of toxicity, none of the compounds are mutagenic for the Ames test, except rhamnocitrin, rutin, and kaempferol. Furthermore, the selected compounds could not inhibit the human ether related gene channel (HERG) and they are not dangerous for cardiovascular patients.

Regarding the elimination pathway, rutin, hispidulin, and ellagic acid have low clearance, while naringenin, (−)-EGC, and (−)-epicatechin gallate demonstrate high clearance. Additionally, all compounds have a short half-life (Bouakkaz et al., 2024b).

### 3.5. Biological activity prediction (PASS)

**Figure 7:**
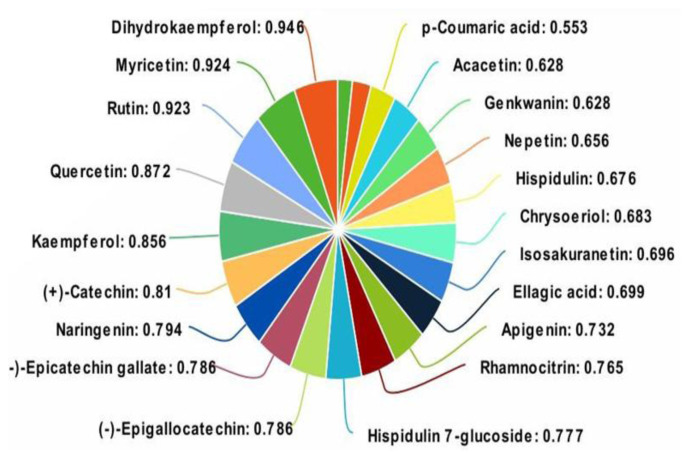
Probability of activity using antioxidant keyword results of selected compounds from *C. villosus* L. var. *undulatus*.

Using the PASS web server, which can be accessed at the URL http://www.pharmaexpert.ru/passonline/, we conducted a thorough analysis of the predicted biological activity (PBA) of specific compounds. This particular web-based tool offers an extensive range of capabilities in terms of predicting various biological activities, encompassing over 1500 distinct categories. These categories encompass a wide array of pharmacological effects, mechanisms of action, toxic and adverse effects, and interactions with metabolic enzymes and transporters, as well as the impact on gene expression. In Figure 7, we provide a concise overview of the PASS results pertaining to the best Pa values. Our meticulous investigation has brought to light that dihydrokaempferol exhibited a remarkable PBA value of 1471, accompanied by a Pa range of 0.924. Conversely, *p*-coumaric acid showed a PBA value of 2453, with the lowest value of the Pa range being 0.553. Astonishingly, all of the selected molecules demonstrated the utmost antioxidant activity, thereby reinforcing their prominence in this regard.

### 3.6. Molecular docking

The results obtained from the molecular docking examination of the first target (PDB ID 2flu) demonstrate that 2,5-dihydroxybenzoic acid and *p*-coumaric acid (phenolic acids) bind to the active site of the enzyme with an energy value of −6.47 and −6.3 kcal/mol, respectively. The hydroxyl groups of these compounds participate in the formation of three hydrogen bonds. For 2,5-dihydroxybenzoic acid, the hydroxyl group forms hydrogen bonds with Ala510, Val604, and Arg415, while, in the case of *p*-coumaric acid, the hydroxyl group forms hydrogen bonds with Val463, Gly367, and Val606. Additionally, Ala366 interacts with the benzene ring of *p*-coumaric acid, forming a hydrophobic interaction of the π–alkyl type. On the other hand, hispidulin (monomethoxy flavone), which is a tricyclic compound, demonstrated a binding affinity of −6.9 kcal/mol. Three hydrogen bonds are observed with this ligand, two of them are formed with the two amino acids Gln530 (2.72 Å) and Tyr334 (1.96 Å), while the third one is formed between the carbonyl and the hydroxyl group of the same inhibitor. Ala556 participated in the formation of two hydrophobic interactions of the π–alkyl type with the A and C ligands’ rings (when A and C are conventional names of flavonoid rings). (+)-Catechin (flavanol), (−)-EGC (flavan-3-ol), and rhamnocitrin (monomethoxy flavone) exhibit the same energy value of −7.3 kcal/mol. Asn382 formed a conventional hydrogen bond with the hydroxyl group of (−)-EGC and rhamnocitrin. Arg380 formed a π–cation interaction with the aromatic ring, specifically, the A ring of (−)-EGC and the B ring of rhamnocitrin. However, it formed a hydrogen bond with the hydroxyl group of ring C in the case of (+)-catechin. Two supplementary hydrogen bonds were found for (+)-catechin with Tyr334 and Gln530. The remaining amino acids contributed to the formation of hydrophobic interactions. Myricetin (flavonol), dihydrokaempferol (flavanonol), and acacetin (monomethoxy flavone) are flavonoids that exhibit the same binding affinity to this target of −7.7 kcal/mol. Arg415, Arg380, and Ser508 participated in the establishment of three hydrogen bonds with lengths of 2.30 Å, 2.71 Å, and 2.73 Å, respectively. Two hydrophobic interaction types, π–π-T-shaped and π–π-shaped, are observed with the aromatic amino acids Tyr334 and Tyr525. Dihydrokaempferol formed two hydrogen bonds: one between the hydroxyl group and the carbonyl group of the inhibitor and the other between the hydroxyl group of cycle B and the hydroxyl group of Tyr334. Furthermore, the aromatic rings A and B created two π–π-T-shaped interactions with Tyr525 (5.44 Å) and Tyr334 (4.96 Å). Furthermore, a single π–cation interaction with Arg380 (4.41 Å) is also recorded with this inhibitor. Acacetin belongs to the flavone class and the presence of this ligand has been noted to result in the formation of two hydrogen bonds with the same length value of 2.89 Å. The first one is observed between the hydroxyl group ported by carbon 5 and Arg415, while the second one is recorded between the methoxy group of cycle C and Asn382. The benzene ring of acacetin participated in the establishment of a π–cation interaction with Arg380 and three hydrophobic interaction types, π–π-T-shaped and π–alkyl, with Tyr334, Ala556, and Tyr525.

The findings for genkwanin, nepetin, and chrysoeriol demonstrated that they bind into the active site of the enzyme with an affinity of −7.01 kcal/mol, −7.1 kcal/mol, and −7.62 kcal/mol, respectively. Their inhibition is the result of the establishment of various interactions. Two conventional hydrogen bonds were recorded in every flavone structure, with Asn382 in the presence of genkwanin and chrysoeriol and with Tyr334 in the presence of nepetin. Ala556 participated in the formation of hydrophobic interactions of the π–alkyl type with both ligands genkwanin and nepetin. Additionally, Tyr334 and Tyr525 formed π–π-T-shaped interactions with genkwanin and chrysoeriol.

(−)-Epicatechin gallate (flavan-3-ol) and hispidulin 7-glucoside are tetracyclic compounds. Compared to the other compounds, the substitution of gallic acid on the C3 of epicatechin and glucosidation in position 7 of hispidulin play a crucial role in the establishment of various interactions. Molecular docking results show that hispidulin 7-*O*-glucoside was bound into the active site of the enzyme with an energy of −7.58 kcal/mol. The carbonyl group and hydroxyl groups interacted with Arg380, Asn382, and Ser555, forming three hydrogen bonds, while the two cycles A and C formed hydrophobic interactions with the aromatic amino acids Tyr572 and Tyr334. On the other hand, (−)-epicatechin gallate formed five hydrogen bonds with Gln530, Arg483, and Arg415. Furthermore, three hydrophobic interactions were recorded with the amino acids Tyr572, Ala556, and Tyr525.

Molecular docking results show that 70% of isosakuranetin (flavanones) poses are docked in the active site of the enzyme, with a binding affinity of −7.98 kcal/mol. A limited number and type of interactions were established with this ligand. One hydrogen bond was formed with Ala556 for isosakuranetin. Additionally, the benzene ring of the ligand participated in the formation of a π–cation interaction with Arg415 and two hydrophobic interactions with Ala556 (5.29 Å) and Tyr572 (3.88 Å).

**Figure 8:**
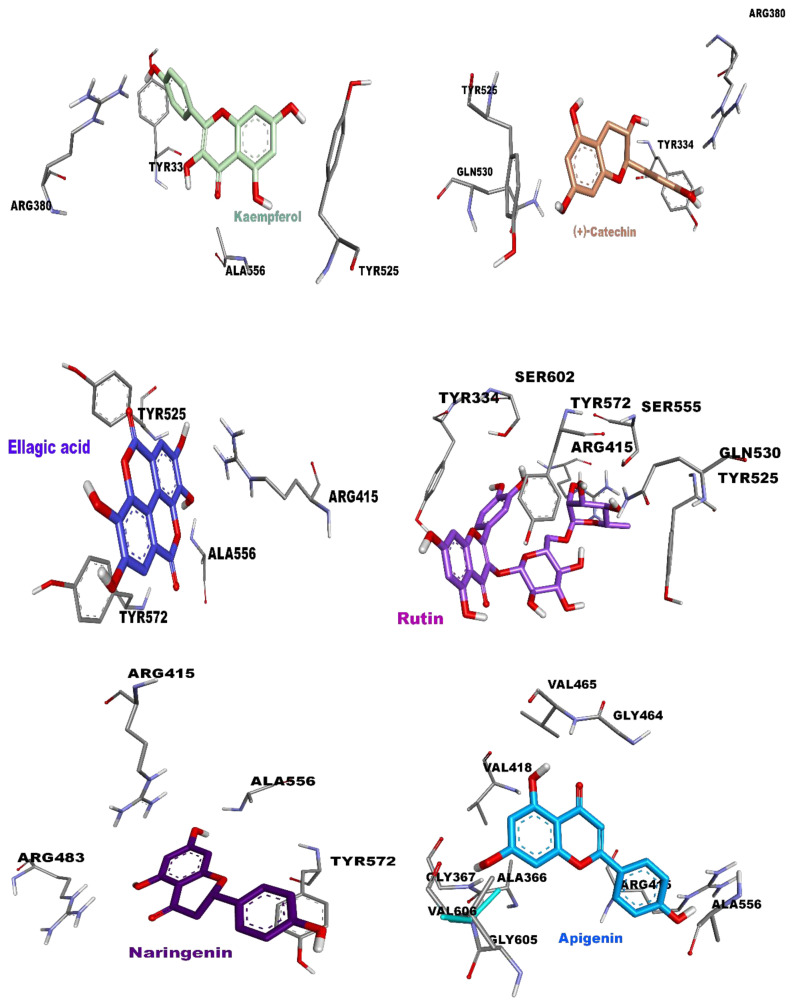
Docking poses of potent inhibitors in the active site of human keap1 (2flu). Inhibitors are shown in different colors, while amino acids of the active site are shown in gray.

Ellagic acid (polyphenol), kaempferol (flavonol), quercetin (flavonol), rutin (flavonoid glycoside), apigenin (flavone), and naringenin (flavanone) were identified as potent inhibitors against human keap1 (2flu), exhibiting energy values less than −8.03 kcal/mol. The hydrophobic interactions observed in these potent ligands were mainly attributed to the presence of Tyr525, Tyr572, Ala556, Tyr334, and Ala366. Moreover, Arg415 formed a π–cation interaction type with ellagic acid and naringenin, whereas it was observed with Arg380 in the case of kaempferol. More precisely, seven important hydrogen bonds were detected in rutin due to its specific conformation, while apigenin formed four hydrogen bonds with Val465, Val606, and Gly367. The remaining potent inhibitors did not exhibit any hydrogen bonds, except for quercetin and naringenin, which displayed a limited number of conventional hydrogen bonds. All the results are summarized in Table 7 and Figure 8.

Among the most potent inhibitors were ellagic acid, kaempferol, quercetin, rutin, apigenin, and naringenin, which formed various hydrogen bonds and hydrophobic interactions with the protein. These findings suggest that these compounds have the potential to disrupt the Keap1–Nrf2 interaction, leading to the activation of the Nrf2 pathway and subsequent antioxidant and cytoprotective effects.

The results obtained from the molecular docking study of the second target (PDB ID: 7yxw) indicated that *p*-coumaric acid and 2,5-dihydroxybenzoic acid were the least potent inhibitors when compared to other inhibitors owing to their uncomplicated structure. They bound with −5.4 kcal/mol and −5.2 kcal/mol, respectively. The carbonyl group of *p*-coumaric acid forms a hydrogen bond with Tyr274 (2.09 Å), while 2,5-dihydroxybenzoic acid forms two hydrogen bonds with Asp261 (2.6 Å) and Tyr167 (2.58 Å). Furthermore, different types of hydrophobic interactions were observed; π–π-T-shaped, π–π-stacked, and π–sigma interactions were recorded with Tyr274 and Trp263 in the presence of *p*-coumaric acid, while π–alkyl and π–π-T-shaped interactions were observed for 2,5-dihydroxybenzoic acid.

(−)-EGC (flavan-3-ol) and myricetin (flavonol) are two flavonoids that exhibit the same energy value of −6.7 kcal/mol. The cycle C hydroxyl group of (−)-EGC formed two hydrogen bonds with Asp261 (1.90 Å) and Gly192 (2.12 Å), whereas myricetin formed four hydrogen bonds with Trp193 (2.61 and 2.86 Å), Tyr279 (2.17 Å), Ser191 (2.40 Å), and Gly192 (2.61 Å). The flavonoid rings participated in the formation of hydrophobic interactions. Tyr167, Pro206, and Trp193 were involved in π–alkyl, π–π-T-shaped and π–π-stacked interactions for (−)-EGC, while Trp263 and Pro206 were responsible for the formation of π–alkyl and π–π-T-shaped interactions with myricetin.

Quercetin (flavonol), acacetin (monomethoxyflavone), hispidulin (monomethoxyflavone), naringenin (flavanone), apigenin (flavone), and nepetin (flavone) were docked with a score of −6.9 kcal/mol, except for apigenin, which bound with −6.8 kcal/mol. All these compounds, with the exception of naringenin, demonstrated the presence of one hydrogen bond of length greater than 2 Å. For nepetin, it interacted with Thr170. Concerning hispidulin, a hydrogen bond is formed between the carbonyl and the hydroxyl group of the same ligand. The different rings of these flavonoids are responsible for the establishment of hydrophobic interaction types. Pro206 and Trp263 formed three hydrophobic interaction types, π–alkyl and π–π-T-shaped, with quercetin. Regarding acacetin and apigenin, they formed four π–π-stacked interactions with Trp193 and one π–π-T-shaped interaction was observed with Tyr167. For the remaining flavonoids, hispidulin, naringenin, and nepetin, it was observed that the most frequent hydrophobic interaction was π–π-stacked, which formed with Trp193 and Pro206.

(+)-Catechin (flavanol), kaempferol (flavonol), chrysoeriol (flavone), genkwanin (flavone), isosakuranetin (flavanone), and rhamnocitrin (monomethoxyflavone) interacted with the active site of 7yxw, with a binding energy of −7 kcal/mol. One hydrogen bond was detected with every ligand except for (+)-catechin, which created two. For kaempferol, it was observed with Trp193 with a length of 2.71 Å, while it was detected with Thr170 in the case of chrysoeriol. Regarding (+)-catechin, genkwanin, and rhamnocitrin, the formation of hydrogen bonds was facilitated by the interaction with Asp261 and Gly192. In addition, the formation of hydrophobic interactions, especially π–π-T-shaped, was recorded for Tyr167 and Trp263, whereas Trp193 was responsible for the formation of π–π-stacked interactions for all ligands. Particularly π–sulfur was saved with (+)-catechin, especially, between the C ring and Met278.

Ellagic acid (polyphenol), hispidulin 7-glucoside, rutin (flavonol), dihydrokaempferol (flavanonol), and (−)-epicatechin gallate (flavan-3-ol) were determined as the strongest tested inhibitors against 7yxw, with energy values lower than −7.1 kcal/mol due to their complex structure, which leads to the formation of various interactions. We noticed that the amino acids Ser191, Gly192, Asp261, Glu244, and Trp193 participated in the formation of hydrogen bonds, two with ellagic acid, hispidulin 7-glucoside, and dihydrokaempferol, while rutin formed four hydrogen bonds. Furthermore, Pro206 exhibited its responsibility in the formation of a π–alkyl interaction, while Trp193, Tyr167, and Trp263 contributed to the formation of the hydrophobic interaction types (π–π-stacked and π–π-T-shaped) present in all these ligands. (−)-Epicatechin gallate established a π–sulfur interaction with Met278.

**Figure 9:**
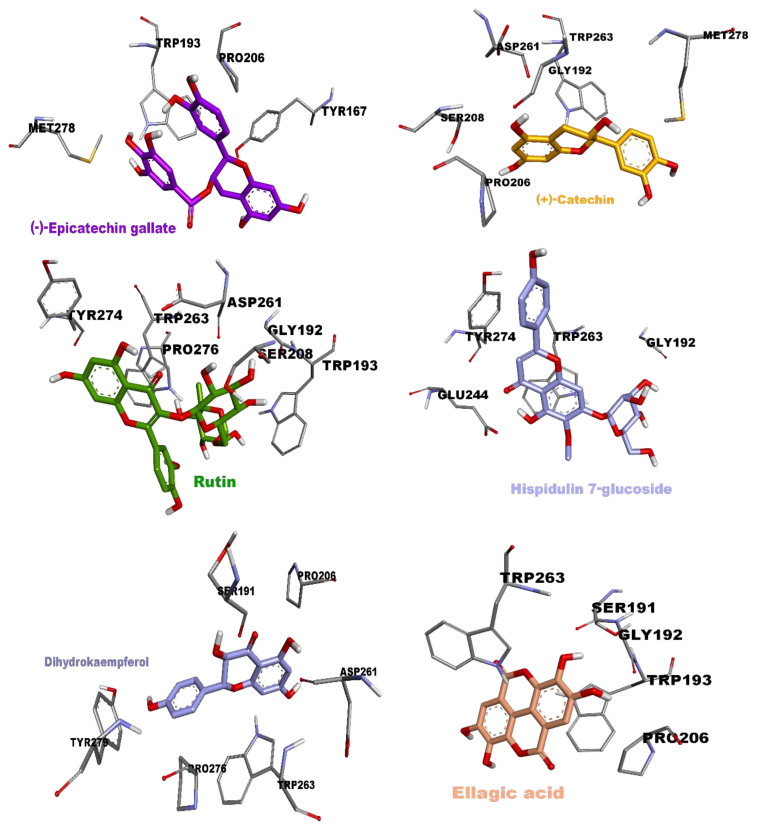
Docking poses of potent inhibitors in the active site of 7yxw. Inhibitors are shown in different colors, while amino acids of the active site are shown in gray.

For all ligands, it docked within the active site of 7yxw with a repetition ratio in the range of 95%–100%. All the results are summarized in Table 8 and Figure 9.

## 4. Conclusion

Our study has unveiled *C. creticus* as a potent natural antioxidant treasure trove, supported by a comprehensive in vitro and in silico investigation. This finding goes beyond the laboratory, as *C. creticus* already holds a place of honor in our traditional practice as a comforting and potentially health-promoting tea. Notably, all identified compounds possess established antioxidant activities, further solidifying its promise as a natural shield against oxidative stress.

The quest for the optimal extraction method led us to maceration, which emerges as the best. Unlike the harsh temperatures of Soxhlet extraction, maceration gently coaxed out the valuable bioactive compounds in their full potency, a fact beautifully corroborated by LC-HRMS profiling of the potent extracts. This study indicates that achieving the desired quantity of phenolic compounds is feasible through the selection of an appropriate extraction method tailored to the specific target.

These findings pave the way for further exploration of *C. creticus*’s potential in promoting human health and well-being. Future research could focus on clinical trials to substantiate its antioxidant and antistress effects in vivo, ultimately leading to the development of safe and effective *Cistus*-based interventions for oxidative stress-related pathologies.

## Figures and Tables

**Table 1:** The grid box parameters for each target.

PDB ID	x*y*z center	x*y*z size
**2flu**	5.424*6.693*2.756	26*32*24
**7yxw**	−22.454*44.753*−9.523	20*26*28

**Table 2:** Extraction yield of the *C. villosus* L. var. *undulatus* extracts.

		Solvent	Extraction yield (%)
**Method 1**	**Maceration**	Hexane	0.44
		Ethyl acetate	0.42
**Method 2**	**Soxhlet**	Hexane	2.27
		Acetone	1.19
		Methanol	13.60

**Table 3:** Qualitative and quantitative analysis of the phenolic compounds of the MeOH extract of *C. villosus* L. by LC-HRMS .^a^

No	Phenolics	Molecular Formula	μg/g extract	U (%)
1	(−)-Epigallocatechin	C_15_H_14_O_7_	1477.88	3.09
2	(−)-Epicatechin gallate	C_22_H_18_O_10_	19.42	3.05
3	Verbascoside	C_29_H_36_O_15_	0.06	2.93
4	Chicoric acid	C_22_H_18_O_12_	3.40	2.28
5	Caffeic acid	C_9_H_8_O_4_	3.66	3.74
6	(+)-*trans* taxifolin	C_15_H_12_O_7_	4.04	3.35
7	*p*-Coumaric acid	C_9_H_8_O_3_	49.00	3.31
8	Rutin	C_27_H_30_O_16_	354.74	3.07
9	Dihydrokaempferol	C_15_H_12_O_6_	8.36	2.86
10	Ellagic acid	C_14_H_6_O_8_	723.60	4.20
11	Myricetin	C_15_H_10_O_8_	150.98	4.18
12	Quercetin	C_15_H_10_O_7_	55.38	2.95
13	Salicylic acid	C_7_H_6_O_3_	51.66	1.89
14	Naringenin	C_15_H_12_O_5_	9.16	4.20
15	Nepetin	C_16_H_12_O_7_	1.12	2.19
16	Kaempferol	C_15_H_10_O_6_	910.10	3.56
17	Apigenin	C_15_H_10_O_5_	3.20	2.87
18	Hispidulin	C_16_H_12_O_6_	4.92	3.41
19	Isosakuranetin	C_16_H_14_O_5_	1.12	3.98
20	Rhamnocitrin	C_16_H_12_O_6_	3.78	3.16
21	Chrysin	C_15_H_10_O_4_	0.12	3.24
22	Acacetin	C_16_H_12_O_5_	12.88	3.98
23	Gentisic acid	C_7_H_6_O_4_	36.18	4.77
24	Hispidulin 7-*O*-glucoside	C_22_H_22_O_11_	426.82	4.57
25	Genkwanin	C_16_H_12_O_5_	0.18	4.44
26	Apigenin 7-*O*-acylglucoside	C_22_H_20_O_11_	1.70	2.70
27	Chrysoeriol	C_16_H_12_O_6_	3.58	2.08
28	Apigenin 7-methylate	C_17_H_12_O_6_	1.36	2.94

a Compared with authentic standard compounds.

**Table 4:** Qualitative and quantitative analysis of the phenolic compounds of the EtOAc fraction of *C. villosus* L. by LC-HRMS.^a^

No	Phenolics	Molecular formula	μg/g extract	U (%)
1	(+)-Catechin	C_15_H_14_O_6_	1739.70	3.31
2	(−)-Epicatechin gallate	C_22_H_18_O_10_	122.10	3.05
3	Verbascoside	C_29_H_36_O_15_	1.72	2.93
4	Caffeic acid	C_9_H_8_O_4_	18.40	3.74
5	(+)-*trans* taxifolin	C_15_H_12_O_7_	16.86	3.35
6	*p*-Coumaric acid	C_9_H_8_O_3_	187.02	3.31
7	Rutin	C_27_H_30_O_16_	1003.16	3.07
8	Rosmarinic acid	C_18_H_16_O_8_	14.68	3.77
9	Dihydrokaempferol	C_15_H_12_O_6_	20.86	2.86
10	Ellagic acid	C_14_H_6_O_8_	868.98	4.20
11	Myricetin	C_15_H_10_O_8_	304.92	4.18
12	Quercetin	C_15_H_10_O_7_	173.66	2.95
13	Salicylic acid	C_7_H_6_O_3_	78.10	1.89
14	Naringenin	C_15_H_12_O_5_	137.26	4.20
15	Nepetin	C_16_H_12_O_7_	46.04	2.19
16	Kaempferol	C_15_H_10_O_6_	118.86	3.56
17	3’-*O*-Methyl quercetin	C_16_H_12_O_7_	8.06	3.58
18	Apigenin	C_15_H_10_O_5_	124.44	2.87
19	Hispidulin	C_16_H_12_O_6_	172.24	3.41
20	Isosakuranetin	C_16_H_14_O_5_	36.48	3.98
21	Rhamnocitrin	C_16_H_12_O_6_	56.06	3.16
22	Chrysin	C_15_H_10_O_4_	0.74	3.24
23	Acacetin	C_16_H_12_O_5_	920.68	3.98
24	Gentisic acid	C_7_H_6_O_4_	71.78	4.77
25	Hispidulin 7-*O*-glucoside	C_22_H_22_O_11_	1656.38	4.57
26	Genkwanin	C_16_H_12_O_5_	81.98	4.44
27	Luteolin-7-*O*-acylglucoside	C_22_H_20_O_12_	52.22	2.12
28	Chrysoeriol	C_16_H_12_O_6_	93.06	2.08
29	Apigenin 7-methylate	C_17_H_12_O_6_	89.18	2.94

a Compared with authentic standard compounds

**Table 5:** Antioxidant activity results of *Cistus villosus* L. var. *undulatus* extracts.

	DPPH	ABTS	CUPRAC	Metal chelating	*b*-carotene
	IC_50_(μg/mL)	IC_50_(μg/mL)	A0.5 (μg/mL)	IC_50_ (μg/mL)	IC_50_(μg/mL)
MCECc	8.19±0.45	1.41±0.45	13.27±0.17	1619.03±24.11	10.45±0.06
ECECc	5.30±0.14	5.10±0.11	8.74±0.19	3035.58±74.19	2.44±0.11
BHT	-	21.67±0.87	26.85±1.50	-	-
BHA	28.59±0.06	7.23±0.01	24.49±0.19	-	1.34±0.04
α-Toc	36.35±0.24	27.70±0.28	134.53±0.19	-	2.10±0.08
EDTA	-	-	-	26.85±1.50	-

The best values are highlighted in bold.

**Table 6:** ADMET results of determined major compounds of *Cistus villosus* L. by LC-HRMS profiling.

Pharmacokinetics	Ellagic acid	Kaempferol	Quercetin	Rutin	Apigenin
**Absorption**					
**Water solubility (mg/L)**	922.46	127.30	-	217.20	159.88
**Caco-2 cell permeability (nm/s)**	20.48	9.57	−5.20	7.91	10.54
**Human intestinal absorption (HIA)**	61.39	79.43	0.01	2.86	88.12
**Distribution**					
**BBB penetration (C. brain/C. blood)**	0.32	0.28	0.008	0.02	0.56
**Plasma protein binding (%)**	88.40	89.60	95.49	43.89	97.25
**Metabolism**					
**CYP3A4 substrate**	NO	NO	NO	YES	NO
**CYP2C19 inhibitor**	NO	NO	NO	NO	NO
**CYP2C9 inhibitor**	NO	NO	NO	NO	NO
**CYP2D6 inhibitor**	NO	YES	NO	NO	YES
**CYP3A4 inhibitor**	NO	YES	NO	NO	YES
**Excretion**					
**T ** **_½_** ** (hour)**	0.886	0.905	0.929	0.52	0.856
**CL (mL/min/kg)**	3.724	6.868	8.284	1.34	7.022
**Toxicity**					
**Human hepatotoxicity**	YES	YES	-	YES	YES
**Carcinogens**	NO	NO	-	NO	NO
**HERG inhibition**	NO	NO	-	NO	NO
**Pharmacokinetics**	Naringenin	Hispidulin 7-*O*-glucoside	Dihydrokaempferol	(−)-Epicatechin gallate	(+)-Catechin
**Absorption**					
**Water solubility (mg/L)**	251.40	2.55	319.73	126.98	1.74
**Caco-2 cell permeability (nm/s)**	10.52	9.24	9.56	13.21	5.971
**Human intestinal absorption (HIA)**	87.31	42.14	77.83	40.58	0.096
**Distribution**					
**BBB penetration (C. brain/C. blood)**	0.59	0.03	0.28	0.14	0.025
**Plasma protein binding (%)**	100	62.36	89.67	100	92.06
**Metabolism**					
**CYP3A4 substrate**	YES	YES	NO	NO	YES
**CYP2C19 inhibitor**	NO	YES	NO	NO	NO
**CYP2C9 inhibitor**	NO	YES	NO	NO	NO
**CYP2D6 inhibitor**	NO	NO	NO	NO	NO
**CYP3A4 inhibitor**	YES	YES	NO	NO	NO
**Excretion**					
**T ** **_½_** ** (hour)**	0.774	0.51	0.625	0.923	0.853
**CL (mL/min/kg)**	17.388	2.64	8.968	17.814	17.911
**Toxicity**					
**Human hepatotoxicity**	NO	NO	NO	NO	YES
**Carcinogens**	NO	NO	NO	NO	NO
**HERG inhibition**	NO	NO	NO	NO	NO

**Table 7:** The outcomes of the molecular docking analysis with the human keap1 (PDB ID: 2flu).

Ligand	Repetition ration(%)	Affinity kcal/mol	Closest residues	Interactions type	Number	Length Å
***p*** **-Coumaric acid**	−6.47	40	Gly509, Val463, Gly367, Ala366, Val606	Hydrogen bond	3	>1.89
				π-alkyl	1	4.27

**(+)-Catechin**	−7.3	60	Arg380, Tyr334, Tyr525, Gln530	Hydrogen bond	3	>2.41
				π-π-T-shaped	2	>4.98

**2,5-Dihydroxybenzoic acid**	−6.3	70	Arg 415, Ala510, Val604, Gly462, Ala366, Leu365	Hydrogen bond	3	>2

**Ellagic acid**	−8.03	60	Arg 415, Ala556, Tyr572, Tyr525	π-alkyl,	2	>4
				π-cation	1	4.63
				π-π-T-shaped	1	5.44
				π-π-stacked	3	>1.85

**(−)-Epigallocatechin**	−7.3	50	Tyr572, Asn382, Arg380	Hydrogen bond	1	2.62
				π-cation	1	4.08
				π-π-T-shaped	1	4.78

**Hispidulin 7-** ***O*** **-glucoside**	−7.58	50	Tyr334, Ser 555, Tyr572, Arg380, Arg415, Gln530	Hydrogen bond	4	>2.23
				π-π-T-shaped	1	5.70
				π-π-stacked	1	5.63

**Kaempferol**	−8.31	50	Tyr525, Ala556, Arg380, Tyr334	π-alkyl	1	5.12
				π-π-T-shaped	2	>5.0
				π-cation	1	4.34

**Myricetin**	−7.73	70	Arg380, Tyr334, Arg415, Tyr525, Ser508	Hydrogen bond		
					3	>2.30
					2	>5.0
				π-π-T-shaped		

**Quercetin**	−8.05	70	Ala556, Ser555, Tyr525, Tyr334	Hydrogen bond	2	>2.34
				π-alkyl	1	5.24
				π-π-T-shaped	2	>5.1

**Rutin**	−8.38	100	Arg415, Tyr334, Ser602, Ser572,Gln530, Tyr525, Ser555	Hydrogen bond	7	>1
				π-alkyl	1	4.61
				π-π-T-shaped	1	4.92
				π-π-stacked	1	4.95

**Acacetin**	−7.76	70	Arg415, Arg380, Asn382, Tyr334, Ala556, Tyr525	Hydrogen bond	2	2.89
				π-alkyl	1	5.09
				π-cation	1	4.30
				π-π-T-shaped	2	>5

**Apigenin**	−8.68	50	Arg415, Ala556, Val465, Val418, Ala366, Gly367, Gly605, Val606, Gly464	Hydrogen bond	4	>1.85
				π-alkyl	3	>4.54
				π-sigma	1	3.72

**Chrysoeriol**	−7.62	80	Ser555, Tyr525, Tyr334, Asn382, Arg380	Hydrogen bond π-π-T-shaped	2	2.27
						2.73
					2	>4.97

**Dihydrokaempferol**	−7.71	90	Tyr334, Tyr525, Arg380	Hydrogen bond	2	1.78
				π-π-T-shaped	2	>4.96
				π-cation	1	4.41

**(−)-Epicatechin gallate**	−7.68	90	Tyr572, Ala556, Arg415, Arg483, Tyr525, Gln530	Hydrogen bond	5	>1.51
				π-alkyl	2	>4.93
				π-π-stacked	1	4.34

**Genkwanin**	−7.01	90	Arg380,Asn382, Tyr334, Ala556, Tyr525	Hydrogen bond	2	>2.77
				π-alkyl	1	5.25
				π-π-T-shaped	2	>5.13
				π-cation	1	4.31

**Hispidulin**	−6.9	100	Ser508, Ala556, Gln530, Tyr334	Hydrogen bond	3	>1.96
				π-alkyl	2	>5.05

**Isosakuranetin**	−7.98	70	Arg415, Ala556, Tyr572	Hydrogen bond	1	2.91
				π-alkyl	1	5.29
				π-π-stacked	1	3.88
				π-cation	1	4.61

**Naringenin**	−8.46	50	Arg483, Arg415, Ala556, Tyr572	Hydrogen bond	1	2.37
				π-alkyl	1	5.29
				π-π-shaped	1	3.88
				π-cation	1	4.60
**Nepetin**	−7.1	100	Ser508, Ala556, Tyr334, Arg380	Hydrogen bond	2	>1.80
				π-alkyl	2	>4.99

**Rhamnocitrin**	−7.35	70	Tyr334, Asn382, Arg380, Ala556, Tyr525	Hydrogen bond	2	1.83
						2.66
				π-alkyl	1	5.17
				π-π-shaped	2	>5.15
				π-cation	1	4.30

**Ascorbic acid**	−6.2	75	Ile416, Gly509, Ala510, Gly603	Hydrogen bond	5	>1.92

**BHA**	−6.3	50	Leu365, Ala366, Val418, Val465	Hydrogen bond	3	>2.10
				π-alkyl	1	4.29

**BHT**	−6	60	Ala366, Val606	π-alkyl	2	4.30
						4.63

**Table 8:** The outcomes of the molecular docking analysis with the second target (PDB ID: 7yxw).

Ligands	Affinity kcal/mol	Repetition ration (%)	Closest residues	Interactions type	Number	Length Å
***p*** **-Coumaric acid**	−5.4	95	Trp263, Tyr274	Hydrogen bond	1	2.09
				π-π-T-shaped	1	5.38
				π-π-stacked	2	>4
				π-Sigma	1	3.67

**2,5-Dihydroxybenzoic acid**	−5.2	100	Tyr167, Trp193, Pro206, Asp261	Hydrogen bond π-alkyl π-π-T-shaped	2	2.58
						2.60
					1	4.24
					1	5.05

**Ellagic acid**	−7.2	98	Ser191, Gly192, Trp193, Pro206, Trp263	Hydrogen bond π-alkyl π-π-stacked	2	2.18
						2.39
					1	5.46
					7	>4.5

**(+)-Catechin**	−7	100	Gly192, Asp261, Trp263, Met278, Ser208, Pro206	Hydrogen bond π-Sulfur π-alkyl π-π-T-shaped	2	2.22
						2.66
					1	4.32
					1	5.2
					1	4.85

**(−)-Epigallocatechin**	−6.7	95	Tyr167, Gly192, Trp193, Pro206, Asp261	Hydrogen bond π-alkyl π-π-stacked π-π-T-shaped	2	1.90
						2.12
					1	5.3
					2	>4.31
					1	4.82

**Hispidulin 7-** ***O*** **-glucoside**	−7.3	100	Gly192, Glu244, Trp263, Tyr274	Hydrogen bond	3	>2.94
				π-π-T-shaped	1	5.13
				π-π-stacked	5	>3.7

**Kaempferol**	−7.0	95	Trp193, Pro206, Trp263	Hydrogen bond	1	2.71
				π-alkyl	1	5.35
				π-π-T-shaped	2	>4.58

**Myricetin**	−6.7	97	Ser191, Gly192, Trp193, Pro206, Ser208, Trp263, Tyr279	Hydrogen bond	4	> 2.17
				π-alkyl	1	5.14
				π-π-T-shaped	3	>4.75

**Quercetin**	−6.9	100	Trp193, Pro206, Trp263	Hydrogen bond	1	2.69
				π-alkyl	1	5.36
				π-π-T-shaped	2	>4.58

**Rutin**	−7.7	90	Gly192, Trp193, Ser208, **Asp261**, Trp263, Tyr274, Pro276	Hydrogen bond	4	>1.91
				π-alkyl	3	>4.03
				π-π-T-shaped	1	5.57
				π-π-stacked	4	>3.59

**Acacetin**	−6.9	95	Tyr167, Ser191,Trp193	Hydrogen bond	1	2.21
				π-π-stacked	4	>4.31
				π-π-T-shaped	1	5.19

**Apigenin**	−6.8	95	Tyr167, Ser191, Trp193, Gly262	Hydrogen bond	2.33	1
				π-π-stacked	4	>4.31
				π-π-T-shaped	1	5.17

**Chrysoeriol**	−7.0	98	Tyr167 ,Thr170, Trp193, Pro206	Hydrogen bond	1	2.83
				π-alkyl	2	>5.29
				π-π-stacked	2	>4.21
				π-π-T-shaped	2	>4.88

**Dihydrokaempferol**	−7.1	95	Ser191, Pro206, Asp261, Trp263, Pro276, Tyr279	Hydrogen bond	2	>2.33
				π-π-T-shaped	1	2.69
				π-alkyl	2	>5.34

**(−)-Epicatechin gallate**	−7.4	90	Tyr167, Trp193, Pro206, Met278	π-sulfur	1	5.54
				π-π-stacked	1	4.54
				π-π-T-shaped	3	>5.07
				π-alkyl	2	>4.02

**Genkwanin**	−7.0	95	Tyr167, Trp193, Asp261	Hydrogen bond	1	2.02
				π-π-stacked	4	>4.32
				π-π-T-shaped	1	5.17

**Hispidulin**	−6.9	98	Tyr167, Ser191, Trp193, Pro206, Gly262, Trp263	Hydrogen bond	1	1.78
				π-π-stacked	4	>4.20
				π-π-T-shaped	2	>4.90
				π-alkyl	1	5.25

**Isosakuranetin**	−7.0	100	Tyr167, Ser191, Trp193, Trp263	Hydrogen bond	1	1.98
				π-π-stacked	2	>4.87
				π-π-T-shaped	2	>4.85

**Naringenin**	−6.9	95	Tyr167, Trp193, Trp263	π-π-stacked	2	>4.87
				π-π-T-shaped	2	>4.84

**Nepetin**	−6.9	100	Tyr167, Thr170, Trp193, Pro206, Gly262	Hydrogen bond	1	2.42
				π-π-stacked	4	>4.19
				π-π-T-shaped	1	4.85
				π-alkyl	1	5.26

**Rhamnocitrin**	−7.0	97	Gly192, Pro206, Ser208, Trp263	Hydrogen bond π-π-T-shaped π-alkyl	1	2.30
					2	4.58
						4.77
					1	5.33

**Ascorbic acid**	−5.1	85	Trp193, Asp261, Gly262	Hydrogen bond	4	>2.38

**BHA**	−5.8	100	Trp193, Gly262, Trp263, Met278	Hydrogen bond	1	3.51
				π-alkyl	4	>5.43
				π-Sigma	2	>3.77

**BHT**	−5.7	95	Trp263, Tyr274	π-π-stacked	2	>4.40
				π-alkyl	5	>4.77
				π-Sigma	1	3.79
